# Olfactory learning in *Pieris brassicae* butterflies is dependent on the intensity of a plant-derived oviposition cue

**DOI:** 10.1098/rspb.2024.0533

**Published:** 2024-08-07

**Authors:** Dimitri Peftuloglu, Stefan Bonestroo, Roos Lenders, Hans M. Smid, Marcel Dicke, Joop J. A. van Loon, Alexander Haverkamp

**Affiliations:** ^1^ Laboratory of Entomology, Wageningen University & Research, Wageningen 6708PB, The Netherlands

**Keywords:** olfactory conditioning, oviposition stimulus, glucosinolates, lepidoptera, *Pieris brassicae*

## Abstract

Butterflies, like many insects, use gustatory and olfactory cues innately to assess the suitability of an oviposition site and are able to associate colours and leaf shapes with an oviposition reward. Studies on other insects have demonstrated that the quality of the reward is a crucial factor in forming associative memory. We set out to investigate whether the large cabbage white *Pieris brassicae* (Linnaeus) has the ability to associate an oviposition experience with a neutral olfactory cue. In addition, we tested whether the strength of this association is dependent on the gustatory response to the glucosinolate sinigrin, which is a known oviposition stimulus for *P. brassicae*. Female butterflies were able to associate a neutral odour with an oviposition experience after a single oviposition experience, both in a greenhouse and in a semi-natural outdoor setting. Moreover, butterflies performed best when trained with concentrations of sinigrin that showed the strongest response by specific gustatory neurons on the forelegs. Our study provides novel insight into the role of both gustatory and olfactory cues during oviposition learning in lepidopterans and contributes to a better understanding of how these insects might be able to adapt to a rapidly changing environment.

## 1. Introduction

All insects rely on innate as well as learned associations between sensory information and behavioural outcomes to survive and thrive in a given environment [1]. Butterflies in particular strongly rely on innate gustatory cues to detect suitable host plants for their offspring. However, changes in the environment expose all animals to challenges that require rapid adaptation, and associative learning has often been proposed to play a key role in this process [2]. Despite the relatively small size of the insect brain, with about 200 000 neurons in fruit flies to about 1 000 000 neurons in honey bees [3,4], insects have been shown to possess great capacities for learning and memory formation [1,5]. These abilities often have positive effects on the survival of individuals and their offspring, since the capability of associating previously neutral stimuli with resource availability might allow them to better find food and locate suitable oviposition sites [6,7]. The ecological factors that may facilitate certain learning abilities have been investigated in a range of insect orders, including Hymenoptera [8–10], Diptera [11], Orthoptera [6] and Lepidoptera [12], demonstrating a high diversity of memory traits [13].

In Lepidoptera, learning and memory have been studied for a range of ecologically relevant tasks, including selection of food sources and oviposition sites [1,7,14–16]. The pipevine swallowtail butterfly, *Battus philenor*, for example, could be conditioned to non-host plants of a certain leaf shape when these had been combined with a plant extract from its host plant during training [17]. In addition, negative experiences with a host plant can alter innate preferences and lead to changes in the decision-making processes of female lepidopterans. An example of this was found in *Danaus plexippus* females, where individuals learned to avoid plants that conspecifics had already laid eggs on and adjusted their egg-laying strategies [7]. Besides these learning assays, visual learning has been investigated in great detail in different butterflies, revealing that butterflies are able to associate colours with an oviposition reward [15], nectar reward [14] or both [16]. At the same time, olfactory learning in butterflies has been tested in a number of studies for flower volatiles [18–20], and it has been demonstrated that butterflies are capable of learning to associate floral odours with sucrose rewards during foraging. However, there is still a considerable knowledge gap with regard to oviposition behaviour, especially for evolutionary comparisons with different moth species in which olfactory learning has been investigated in some detail [21,22].

During associative learning, an innately meaningful stimulus called the unconditioned stimulus, is associated with a neutral cue, such as a certain odour or colour, called the conditioned stimulus [23]. For instance, honeybees, but also butterflies, can be trained to extend their proboscis in response to a given odour (conditioned stimulus) when this is followed with a sucrose reward (unconditioned stimulus). After repeated experiences, individuals will learn to associate conditioned and unconditioned stimuli and ultimately respond with a proboscis extension also upon exposure to the conditioned stimulus [19,24]. In parasitoid wasps as well as moths and butterflies, oviposition has been used as a reward during associative learning [17,25,26]. For both food and oviposition learning, the strength of the unconditioned stimulus has often been found to be of critical importance for the formation of short- or long-term memory [27,28]. The term strength, in this case, refers to the intensity with which a given stimulus is detected by the organism’s sensory system. However, in many study systems it has been difficult to precisely quantify the strength of the unconditioned stimulus, especially in relation to oviposition learning [25].

The lepidopteran family Pieridae includes several species whose larvae feed on plants containing glucosinolates, a group of compounds mostly found in Brassicaceae, but also in plants belonging to the Tropaeolaceae, Capparaceae and Resedaceae [29]. In *Pieris* butterflies, host-plant suitability is determined using different sensory modalities. A potential host plant is initially assessed by visual [30,31] and olfactory cues [32]. Upon landing, a female will perform a behaviour known as ‘drumming’: a rapid series of foreleg taps on the leaf surface [31] (see electronic supplementary material, video). This behaviour allows tarsal chemosensory sensilla to sample the leaf cuticle and detect the presence of glucosinolates, enabling a female to evaluate a plant as a suitable host for its offspring [33]. Previous research has found that the glucosinolates glucobrassicin and sinigrin induce oviposition behaviour in *Pieris brassicae*, even when sprayed on a green paper disc [34].

Electrophysiological recordings with *P. brassicae* and *Pieris rapae* have suggested the presence of at least two different gustatory receptor neurons in trichoid sensilla on the fifth tarsomere of the adult butterfly that are involved in the detection of these two oviposition stimuli [35,36]. In *P. brassicae*, these tarsal chemosensory sensilla occur in higher numbers in females than in males, suggesting an involvement in host-plant recognition and oviposition [35]. In *P. rapae*, these sensory organs have been divided into two clusters, namely medial and lateral, according to their position in relation to the leg’s longitudinal axis [36]. Previous electrophysiological recordings in response to glucosinolates and other organic compounds highlighted different responses between the two clusters, with medial sensilla showing neuronal responses to aliphatic (e.g. sinigrin), indolic and aromatic glucosinolates, while for the lateral sensilla responses have only been observed for indolic and aromatic glucosinolates [37]. In *P. rapae*, *PrapGR28* has been identified as the gene coding for the main receptor for sinigrin detection [37], and a direct homologue of this gene has also been identified in *P. brassicae*, suggesting a similar detection mechanism for sinigrin in both species [38].

For these two *Pieris* species, the detection of sinigrin has also been shown to function as an unconditioned stimulus during associative learning, meaning that this glucosinolate is perceived as a reward by an ovipositing female since its detection helps the females to assess the suitability of a host plant for the development of its offspring [15,39]. In this context, higher concentrations of sinigrin are expected to be perceived as more rewarding than lower ones, and it might, therefore, be possible to directly measure the perceived strength of the unconditioned stimulus during oviposition learning by training the butterflies with different sinigrin concentrations.

In this study, we hypothesized that there is a direct relationship between the strength of the neuronal response to different sinigrin concentrations and the behavioural response of butterflies conditioned with a corresponding concentration of sinigrin as oviposition stimulus. Butterflies were trained to an odour as conditioned stimulus with different concentrations of sinigrin on green paper as unconditioned stimulus, and their memory retention was tested after 1 h of consolidation time under both greenhouse and semi-natural conditions. Additionally, we performed electrophysiological recordings on tarsal chemosensory sensilla to screen for neural activity in response to different sinigrin concentrations. Through this approach, we aim to deepen our understanding of the ecological and evolutionary role of oviposition learning during host-plant selection in butterflies.

## 2. Material and methods

### (a) Insects

*Pieris brassicae* (Linnaeus) butterflies were reared in the Unifarm greenhouse facilities of Wageningen University & Research, Wageningen, the Netherlands, at a temperature of 22° ± 2°C and at relative humidity levels of approximately 55%. Caterpillars were feed with Brussels sprouts plants, *Brassica oleracea* var. gemmifera cv. Cyrus under a light: dark cycle of 14:10 h until pupation. After eclosion, butterflies were transferred to a separate cage, in an approximately 1 : 1 male : female ratio and with no contact with host plants. Food was provided in the form of a 10% sucrose/water solution. All butterflies used in this study were tested three days after eclosion in order to standardise physiological states, as well as to ensure that females were mated and therefore prone to oviposit.

### (b) Butterfly conditioning

Butterfly conditioning was performed both in an indoor and outdoor setting. Mated adult females of *P. brassicae* were conditioned by presenting them with a green paper disc (Trophée 1224 ‘Forest Green’, Clairefontaine, France), 9.5 cm in diameter, placed on a wooden stick. This disc was impregnated with 1 ml of sinigrin solution (unconditioned stimulus) at the concentration of interest (either 10-, 20- or 30 mM sinigrin diluted in distilled water) using a chromatographic sprayer (Desaga, Germany). The sinigrin disc was connected to a 1.44 cm^2^ filter paper square, which was clamped about 1.5 cm above the disc with the aid of iron wire. On this filter paper, 20 µl of vanilla extract (30% ethanol extract, Royal Brand Bourbon Vanilla extract; Nielsen-Massey Vanillas Intl., the Netherlands) was loaded to act as a conditioned stimulus. The whole structure was held upright by placing the stick into a plant pot containing sand. The disc was then placed into a textile cage (60 × 60 × 90 cm, Vermandel, the Netherlands) with several female butterflies, each individually numbered on the ventral side of the forewings with a permanent marker. Sucrose solution (10% in water) was made available *ad libitum*. After a successful oviposition experience (i.e. completion of an oviposition event without disturbance, independent of the number of eggs laid), females were transferred individually to a separate cage (40 × 40 × 60 cm, Vermandel, the Netherlands), where sucrose solution was also made available *ad libitum*, to facilitate uninterrupted memory consolidation. In the case of the indoor environment, the butterflies were trained in the same greenhouse compartment at the Unifarm facilities of Wageningen University, which was also used for behavioural tests. For the outdoor training, the cages were set up next to the flight tent at the Droevendaal farm of Wageningen University, described in the next section. At the beginning of each testing day, a fraction of the three days old mated female butterflies were kept in a separate cage, without undergoing any conditioning. These control animals had not been exposed to any sinigrin, green paper discs or vanilla scent during the three days after eclosion, while sucrose solution was provided *ad libitum* until testing. Assessing the response of these naive control animals without an oviposition experience allowed us to establish the innate response of the butterflies towards the green paper discs in combination with the vanilla scent, which could then be compared to the response of the animals trained with different concentrations of sinigrin.

### (c) Behavioural tests

Similar to the conditioning, behavioural tests were conducted in both indoor and outdoor settings to assess whether behavioural responses are consistent across different levels of environmental complexity. For indoor testing, a 1.75 × 1.75 × 1.75 m cage (Vermandel, the Netherlands) was set up in the Unifarm greenhouse facilities of Wageningen University (electronic supplementary material, figure S1*a*). These experiments were conducted between October 2021 and April 2022. Temperature ranged between 24°C and 29°C. Relative humidity was kept at approximately 55%. Illumination for the indoor set-up was provided by both natural sunlight and high-pressure sodium lamps, and light intensity ranged from 5533 to 26 170 Lux, while barometric pressure varied between 998 and 1041 hPa. All environmental conditions, such as light intensity, temperature, relative humidity, wind speed and barometric pressure were recorded before every test (Luxmeter: LX−10, Voltcraft, Germany; weather station: EFWS 01002, Eurochron, Germany).

For outdoor testing, a 12 × 12 × 2.5 m mesh-tent (electronic supplementary material, figure S2*a*) was set up at the Droevendaal farm of Wageningen University (51°59'38.9"N 5°39'38.0"E). Experiments were conducted from June to September 2021 and from April to August 2022. Environmental conditions were measured in a similar way as in the indoor set-up. Light intensity ranged from 15 470 to 82 900 Lux, temperature from 18° to 33°C, relative humidity from 38 to 86%, wind speed from 0 to 1.2 m s^−1^ and barometric pressure from 1010 to 1029 hPa. The vegetation in the flight-tent and in the close surroundings consisted of grasses such as Poaceae, Juncaceae and Cyperaceae and herbaceous plants like *Bellis perennis*, *Rumex acetosa*, *Trifolium pratense, Trifolium repens* and *Taraxacum officinale*, which are typical species in the natural habitat of *P. brassicae* in the Netherlands [40]. During the experiments, the vegetation in the flight tent was maintained at a height range between 10 and 30 cm. Flowers of *Trifolium* spp. and *T. officinale* were available in the tent at all times, and butterflies were allowed to forage freely during the test period. A diversity of insects and other invertebrates have been observed in the tent over the course of the field season. Behavioural observations in both the outdoor setting and greenhouse were carried out between 10.00 and 16.00 h.

The testing environment in both settings contained four pots with wooden sticks and green paper discs not impregnated with sinigrin (electronic supplementary material, figure S1*a* and S2*a*,*b*). In the indoor setting, these pots were set about 1.5 m apart and in the outdoor setting these were about 8 m apart. Two of the discs would bear a filter paper square scented with vanilla extract, while the remaining two would bear a filter paper square impregnated with the solvent as a control solution (30% ethanol/water solution, similar to the solvent of the vanilla extract). After each subsequent test, the position of the discs was rotated in a clockwise manner, to eliminate directional bias.

In addition to direct observations, we monitored the activity on the discs using a multi-channel camera set-up consisting of four individual cameras and a hard disc data recorder (Cabled 4-dome-system PLUS, Bascom Cameras, the Netherlands). For the greenhouse, the cameras were placed at the midline of the cage on an aluminium frame, with two cameras monitoring the discs from above and two cameras observing the set-up from the side. In the outdoor set-up, cameras were mounted about 1.8 m above the four discs on the tent frame. Video recordings were used to confirm the direct behavioural observations when needed.

Once the test environment was prepared, abiotic parameters such as temperature, light intensity and barometric pressure were measured, and a single butterfly was released with the aid of a small launching cage (30 × 30 × 30 cm, Vermandel, Netherlands) placed at the centre of the flight tent. Each animal was given a total of 15 min to exit the cage, upon which recording started. Behaviour was observed for 30 min, during which the time spent on each disc and the total number of visits to each disc, as well as the number of eggs laid, were recorded.

### (d) Electrophysiological tests

To test for a possible relationship between memory retention and intensity of the chemosensory response, recordings of tarsal taste neuron activity were performed on the foreleg medial and lateral sensilla (figure 3*a,b*). Butterflies used for electrophysiological recordings were three days old mated females, reared under the same conditions as those for the behavioural tests. Four concentrations (1, 10, 20 and 30 mM) of sinigrin (Sigma-Aldrich, Germany) in 1 mM KCl were tested. The sinigrin concentrations used in our study cover a range also tested in other studies [37], in addition we included higher concentrations to explore the full range of the response curve of *P. brassicae* for this compound. A KCl solution was used as a blank control at the beginning of each recording round. For each animal tested, one medial and one lateral sensillum were screened for responses to sinigrin, chosen randomly from within one of the four clusters found on the fifth tarsomere (figure 3*a*) [35,36].

#### (i) Stimulus and insect preparation

Glass capillaries (thin wall, 4″, with filament, 1.5 mm; World Precision Instruments, USA) were prepared using a micropipette puller (model P-1000, Sutter Instruments, USA) resulting in a tip opening of approximately 3 µm. For each stimulus, a glass capillary was filled with 20 µl of the solution. Insects were prepared for recordings by removal of the head, wings, mid- and hindlegs and were mounted on a microscope slide covered with double-sided tape. To limit body movement the preparation was wrapped with Parafilm. Subsequently, the forelegs were extended in a forward position and fixed in place on the microscope slide using moulding clay to facilitate access to the tarsal sensilla. Lastly, the body was connected to an electrical ground through a silver wire inserted in the insect’s thorax.

#### (ii) Tarsal taste recordings

A two-minute time interval between the two recordings was maintained. Action potentials were measured using a high impedance probe, amplified using a 10× pre-amplifier (TastePROBE, Ockenfels Syntech, Germany) with lower and higher frequency limits of 100 to 3000 Hz and finally transformed using an analogue-to-digital converter (IDAC4, Ockenfels Syntech, Germany). Responses were visualized and recorded on a PC at a sampling rate of 11 904 bits per second using the software AutoSpike (version 3.9, Ockenfels Syntech, Germany). The total number of spikes was counted for the first second after stimulation onset, and these were divided into two different categories based on amplitude: spikes with an amplitude greater than 1 mV were considered ‘large spikes’, while those with an amplitude between 0.5 and 1 mV were considered ‘medium spikes’ (figure 3*c*). These two spike categories correspond to the neural activity of two different gustatory neurons housed in the tarsal sensilla and have been described in several previous studies in *P. brassicae*, *P. rapae* and *Pieris napi* [35–37,41]. Spikes with an amplitude lower than 0.5 mV occurred with a low frequency (median: 6 spikes s^−1^) and were at times indistinguishable from recording artefacts, therfore these were excluded from the analysis.

### (e) Scanning electron microscopy

Legs were collected from newly emerged adult female *P. brassicae* and fixed overnight at 4°C in 2.5% glutaraldehyde in a 0.1 M cacodylate buffer. Subsequently, samples were rinsed in 0.1 M cacodylate buffer dehydrated using increasing concentrations of ethanol: 30, 50, 70, 90 and 96% ethanol for 1 min and finally two times in 100% ethanol for 5 min. Afterwards, samples were critical point dried (Leica EM CPD300, Germany) to preserve the surface structure of samples. Finally, samples were mounted on stubs and coated with tungsten.

Images were obtained using a FEI Magellan 400 scanning electron microscope. Tarsal sensilla were identified and scanned at magnifications of 250 × (overview) and 25 000 × (sensilla pore) and at an accelerating voltage of 2 kV and a beam current of 13 pA.

### (f) Statistical analysis

All analyses were performed in R (version 4.4.0; R Core Team, 2016) in combination with RStudio (Boston, USA). For the behavioural data, preference indices (PI) for both residence time on vanilla-scented discs and number of visits to the vanilla-scented discs were calculated. In order to do so, for each tested animal, the time spent on unscented discs was subtracted from the time spent on the vanilla-scented discs and the difference was divided by the total time spent on both disc types. Similarly, the number of visits to the unscented discs was subtracted from the number of visits to the scented discs, and the difference was divided by the total number of visits. This approach was taken for all four experimental treatments, and results were analysed with a generalized linear model (GLM) with a beta-binomial (PI—time spent) or a binominal (PI—visits) data distribution using the package glmmTBB [42]. Environmental parameters (indoors: temperature, light intensity and barometric pressure, while wind speed and relative humidity did not vary and were not included; outdoors: temperature, light intensity, wind speed, barometric pressure and relative humidity) were included as additional factors besides the experimental treatment. Estimated marginal means of the different sinigrin treatments were compared in a Tukey-corrected post-hoc analysis (package: emmeans [43]). To test for potential learning and memory recall within each group, PIs for each treatment were also tested for a significant difference from zero through a Wilcoxon signed-rank test. In addition, the time spent on the two vanilla-scented discs was summed, as was done for the time spent on the two unscented discs, and results were divided by the total duration of the behavioural observation. For each experimental treatment, pairwise comparisons between total time spent on the scented and unscented discs were performed; given the non-normal distribution of the data, Wilcoxon signed-rank tests were used. Oviposition instances on scented *vs*. unscented discs were compared for all treatments using a Fisher’s exact test. For the electrophysiology data, while the assumption of sphericity (Mauchly’s test) was met in most cases, the response variables were not normally distributed for the different concentrations (Shapiro–Wilk test) for all spike and sensilla types. Thus, the electrophysiological data was transformed using aligned rank transform, facilitated by the ARTool Package [44], to be used in a linear mixed model with the random effect being butterfly ‘id’, as serial recordings from the same sensillum were repeated measures. For these data, estimated marginal means were compared in a Tukey-corrected post hoc analysis (package: emmeans [43]).

## 3. Results

### (a) Behavioural tests—indoor trials

**Figure 1.**
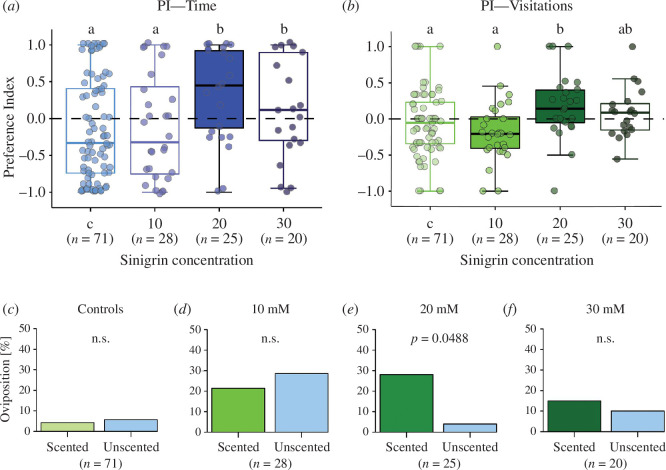
The effect of training with different concentrations of an unconditioned oviposition stimulus (sinigrin) on olfactory learning of mated *Pieris brassicae* females in an indoor setting. (*a*) Preference index for the time spent on vanilla-scented discs for the four treatments. (*b*) Preference index for the visits to vanilla-scented discs for the four treatments. (*c,d,e,f*) Comparison of the percentage of ovipositing animals for the four treatments. Generalized linear models followed by Tukey-corrected estimated marginal means tests were used to compare treatments in (*a*) and (*b*). Letters indicate significant differences (*p *< 0.05). Preference indices of the different treatments were tested against zero using a Wilcoxon signed-rank test. Filled box plots indicate preference indices significantly different from zero (*p *< 0.05). Fisher’s exact test was used to compare differences in the oviposition events between scented and unscented discs within each treatment in (*c,d,e,f*).

We first set out to assess the influence of the oviposition stimulus strength on the ability of a female butterfly to associate an oviposition experience with a novel odour in a simplified environment. Comparisons between treatments for the PIs calculated for the residence time on vanilla scented discs showed a significant effect of concentration (*χ* ^2^ = 31.0231, *p *< 0.001; figure 1*a*; electronic supplementary material, table S1), with butterflies conditioned on 20 mM and 30 mM sinigrin having a higher PI than control butterflies without prior training and oviposition experience (control−20 mM: *p *< 0.001, control−30 mM: *p* = 0.0011) and than butterflies trained on 10 mM (10 mM–20 mM: *p* = 0.0043, 10 mM–30 mM: *p* = 0.0446). In addition, only the PI of butterflies trained with 20 mM sinigrin was significantly different from zero indicating a preference for vanilla-scented discs (*V* = 255.5, *p* = 0.0128; figure 1*a*). Besides concentration also temperature had a significant positive effect on the residence time PI (*χ* ^2^ = 13.3936, *p* = 0.0003; electronic supplementary material, table S1) in the way that higher temperatures lead to higher PI values; however, no interaction between the sinigrin concentration and temperature was observed. The PIs calculated on the number of visitations to vanilla scented discs also revealed a significant effect of concentration (*χ* ^2^ = 11.6530, *p* = 0.0087; figure 1*b*; electronic supplementary material, table S2) with a significant difference between control butterflies and butterflies conditioned on 20 mM sinigrin (*p* = 0.0336) as well as between butterflies conditioned on 10 mM and those conditioned on 20 mM sinigrin (*p* = 0.0084). Comparisons against zero showed significance for both 10 mM and 20 mM trained butterflies, with 10 mM trained butterflies having a preference for unscented discs and 20 mM trained butterflies having a preference for scented discs (10 mM: *V* = 80.5, *p* = 0.0163; 20 mM: *V* = 192, *p* = 0.0347). For the PI based on the number of visits, no significant effect of environmental parameters was observed (electronic supplementary material, table S2). Moreover, we conducted pairwise comparisons for the proportion of time spent on scented and unscented discs for all butterflies trained with different concentrations of the oviposition stimulus. These comparisons revealed that control butterflies, which had no prior experience with the discs or the vanilla scent, spent significantly more time on unscented discs (*V* = 830, *p* = 0.0242; electronic supplementary material, figure S1*b*), while butterflies conditioned with 20 mM sinigrin as unconditioned stimulus spent significantly more time on vanilla scented discs (*V* = 247, *p* = 0.0238; electronic supplementary material, figure S1*d*). Butterflies trained with 10 mM or 30 mM did not differ significantly in their time spent on scented or unscented discs (electronic supplementary material, figure S1*c,e*). Lastly, comparisons for oviposition instances on vanilla-scented versus unscented discs indicated that for butterflies conditioned on 20 mM sinigrin the number of oviposition events on vanilla-scented discs was significantly higher than on unscented discs (Fisher’s exact, *p* = 0.0488; figure 1*e*), while this was not the case for any other treatment (figure 1*c,d,f*).

### (b) Behavioural tests—outdoor trials

**Figure 2.**
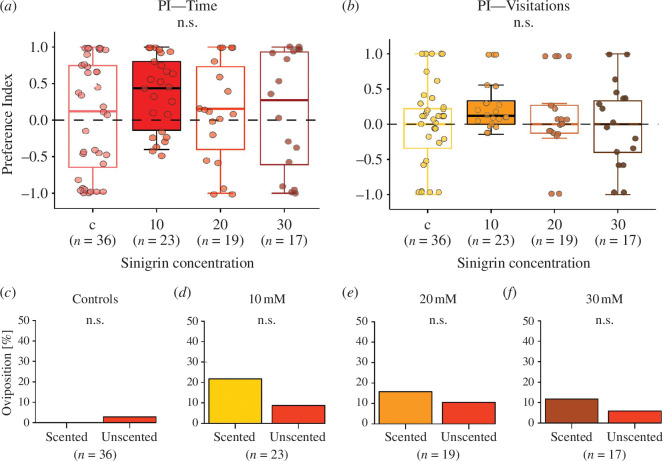
The effect of training with different concentrations of an unconditioned oviposition stimulus (sinigrin), on olfactory learning of mated *Pieris brassicae* females in an outdoor setting. (*a*) Preference index for the time spent on vanilla-scented discs for the four treatments. (*b*) Preference index for the visits to vanilla-scented discs for the four treatments. (*c,d,e,f*) Comparison of the percentage of ovipositing animals for the four treatments. Generalized linear models followed by Tukey-corrected estimated marginal means tests were used to compare treatments in (*a*) and (*b*). Preference indices of the different treatments were tested against zero using a Wilcoxon signed-rank test. Filled box plots indicate preference indices significantly different from zero (*p *< 0.05). Fisher’s exact test was used to compare differences in the oviposition events between scented and unscented discs within each treatment in (*c,d,e,f*).

To test the effect of the oviposition stimulus strength on the memory retention of a female butterfly also in a more complex environment, we first conducted comparisons between treatments for the PI calculated on the residence time on vanilla-scented discs. This comparison did not indicate a significant effect of the sinigrin concentration on butterfly preference (*χ* ^2^ = 3.0023, *p* = 0.3913; figure 2*a*; electronic supplementary material, table S3). However, comparisons of PI values for the time spent on vanilla-scented discs against zero showed that butterflies conditioned with 10 mM had a significant preference for the scented discs (*V* = 278, *p* = 0.0020; figure 2*a*). The PI based on the residence time, was not significantly influenced by any of the environmental parameters (electronic supplementary material, electronic supplementary material, table S3). Similarly, the PI calculated on the number of visitations to vanilla- scented discs did not show any significant difference between treatments (*χ* ^2^ = 1.5885, *p* = 0.6627; figure 2*b*; electronic supplementary material, table S4), but comparisons against zero highlighted again a significant preference of 10 mM trained butterflies for scented discs (*V* = 154, *p* = 0.0030; figure 2*b*). No significant effect of the measured environmental parameters on the PI based on the number of visits was observed (electronic supplementary material, table S4). We also conducted pairwise comparisons for the proportion of time spent on both scented and unscented disc for all treatments in an outdoor arena. These tests show that 10 mM trained butterflies spent significantly more time on vanilla-scented discs (*V* = 265, *p* = 0.0061, electronic supplementary material, figure S2*d*). All other training treatments did not lead to a significant difference in the time spent on either scented or unscented discs (electronic supplementary material, figure S2*c,e,f*). Lastly, comparisons for oviposition instances performed with Fisher’s exact test comparisons for oviposition events on vanilla scented versus unscented discs did not indicate any significant differences (figure 2*c*–*f*).

### (c) Electrophysiology

**Figure 3.**
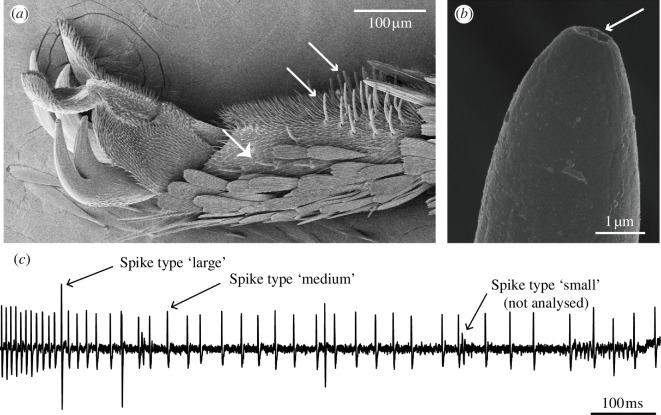
Tarsal taste sensilla and spike classification. (*a*) Fifth tarsomere of the foreleg of a *Pieris brassicae* female. Small arrowheads indicate the two medial sensillum clusters, while the big arrowhead indicates the visible lateral sensillum cluster. Due to view perspective, the other lateral sensillum cluster is not visible. (*b*) Tip of a sensillum in the medial cluster showing a single tip pore (arrow) typical for gustatory sensilla. (*c*) A recording from a sensillum in the medial cluster of the fifth tarsomere stimulated with a 10 mM sinigrin solution. Three different classes of spikes can be distinguished by amplitude. Scale bars for (*a*) and (*b*) can be found in the images.

**Figure 4.**
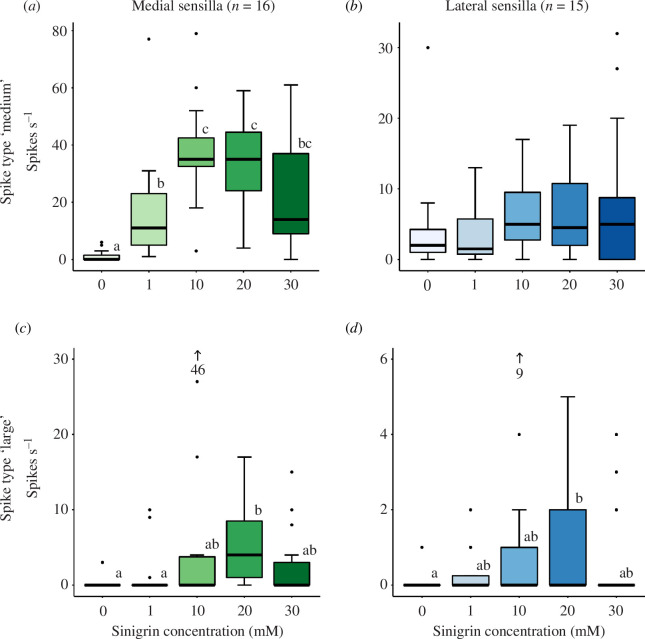
Action potential activity recorded during the first second after stimulation from tarsal taste neurons in sensilla of the medial and lateral clusters to control solution (1 mM KCl; ‘0’) and different concentrations of sinigrin. (*a*) Median firing rate (spikes s^−1^) with an amplitude between 0.5 and 1 mV of neurons in the medial sensilla cluster. (*b*) Median firing rate with an amplitude between 0.5 and 1 mV of neurons in the lateral sensilla cluster. (*c*) Median firing rate with an amplitude higher than 1 mV of neurons in the medial sensilla cluster. (*d*) Median firing rate with an amplitude higher than 1 mV of neurons in the lateral sensilla cluster. Different letters indicate significant differences (*p *< 0.05) in (*a,c*) and (*d*) determined by a mixed-linear model followed by a Tukey-corrected estimated marginal means test. Within one animal (*n* = 16 for medial sensilla and *n* = 15 for lateral sensilla) all concentrations were tested on the same medial and lateral sensillum. For each animal sensilla were chosen randomly from the two clusters.

By measuring the electrophysiological response of taste neurons, we are able to determine the strength of the response to a certain sinigrin concentration by the tarsal gustatory receptor neurons of female butterflies. These electrophysiological responses were divided according to sensillum type and spike amplitude class (figure 3*c*). For ‘medium spikes’, with amplitude between 0.5 and 1 mV, recorded from medial sensilla, a significant effect of concentration was found (*F*_4,56_ = 22.396, *p *< 0.0001; figure 4*a*). Here, the response to the control solution (1 mM KCl) and to 1 mM sinigrin significantly differed from each other (*p *< 0.0001), and were both significantly lower than the responses obtained after stimulation with 10 and 20 mM sinigrin (0–10 mM and 0–20 mM: *p *< 0.0001; 1–10 mM: *p* = 0.0028; 1–20 mM: *p* = 0.0083). No significant difference was found between responses to 1 and 30 mM (*p* = 0.7907), between 10 and 20 mM (*p* = 0.9961) and between 20 and 30 mM responses (*p* = 0.1441). For ‘large spikes’ (amplitude>1 mV), recorded from medial sensilla, a significant effect of concentration was found (*F*_4,56_ = 6.6446, *p* = 0.0002; figure 4*c*), with significant differences being observed between 0 and 20 mM (*p* = 0.0001), and 1 and 20 mM (*p* = 0.0028). For ‘medium spikes’, recorded from sensilla in the lateral clusters, no significant effect of concentration was found (*F*_4,60_ = 1.6038, *p* = 0.18506; figure 4*b*). In the lateral cluster a significant effect of concentration was detected for the ‘large spikes’ (*F*_4,60_ = 2.6512, *p* = 0.0417; figure 4*d*) with 20 mM eliciting a significantly higher response than to the control solution (*p* = 0.0354).

## 4. Discussion

Most lepidopteran species display a high degree of host-plant specialization, and yet their behaviour needs to remain sufficiently flexible to adapt to novel environmental cues [1]. Our results demonstrate that the large cabbage white butterfly, *P. brassicae*, is able to associate a novel odour with a positive oviposition experience within a single trial, both in a greenhouse and in a semi-natural outdoor setting. Moreover, we confirmed that memory recall performance in these environments was dependent on the concentration of a plant-derived gustatory cue, sinigrin, used to induce oviposition during training. The unconditioned stimulus concentration levels that gave higher memory retention were different between the two experimental settings, namely 20 mM for the indoor and 10 mM for the outdoor setting. However, electrophysiological recordings revealed that neuronal responses to both concentrations were equally high, suggesting that these differences are likely due to differences between the two training and testing environments. Finally, we argue that there is a correlation between memory retention and the dose-response curve of the taste neurons on the female tarsi.

In our indoor behavioural observations, we found a pronounced effect of the unconditioned stimulus concentration on the time spent on vanilla-scented discs, the number of visits and the number of eggs laid on these discs, which were all significantly increased in butterflies trained with a 20 mM sinigrin solution. Results from outdoor behavioural observations showed a similar effect of the unconditioned stimulus concentration on memory retention; however, rather at a concentration of 10 mM. One possible reason for this could lie in the different nature of the two training and testing environments. Our indoor environment was relatively stimulus-poor, displaying nothing more than the discs used to assess memory retention, while the outdoor testing environment had a great diversity of sensory stimuli that might have influenced the perception of the oviposition stimulus by the female butterflies. Gustatory neurons in insects generally project to the suboesophageal ganglion and from there to the mushroom bodies in the insect brain [45]. The mushroom bodies receive inputs from different sensory modalities and the association between the rewarding sinigrin stimulus and the novel vanilla odour in our study is likely made by the Kenyon cells in the mushroom bodies. However, as these Kenyon cells also receive further information from the environment, it is possible that the response of these cells was also modified by the input of additional plant odours in the outdoor setting [46]. This might have led to memory formation taking place at a lower sinigrin concentration in the more natural environment.

Although we did find that butterflies learned the novel odour in the outdoor setting already at a lower sinigrin concentration, we also found less pronounced effects of the training with regards to the preference indices and the number of oviposition events in the outdoor environment. These smaller differences might be caused by a shifted attention of the tested animal from searching for oviposition sites to exploratory activities and nectar foraging. In such a scenario, it is likely that learned associations that are not sufficiently strong are forgotten or not acted upon [47]. At the same time, differences in environmental conditions between the two set-ups might have affected the strength of the olfactory stimulus during the memory tests. The stimulus detectability could have been hampered by the presence of other olfactory cues or different climatic factors (i.e. wind or different temperatures). Potentially, these different conditions could have translated into less visits or time spent on the scented discs in the outdoor setting, even though temperature was the only climatic factor that had a significant effect on the butterflies and this also only in the indoor setting where tests were performed in late autumn and early spring. Besides this, our control animals, which did not have an oviposition experience, provide an indication of the innate response of the butterflies to the vanilla-scented discs. These untrained animals showed a significant innate aversion in the indoor set-up, but not in the outdoor set-up, indicating that environmental conditions other than climatic factors, influenced the odour detection and, therefore, also the response of the trained animals to the scented and unscented discs [48]. These observed differences in experimental settings might explain why we did see stronger memory retention in indoor settings, while outdoors these were, in some cases, less pronounced.

Regardless of these differences, the highest degree of memory retention was observed for animals conditioned on sinigrin concentrations of 10 and 20 mM, and these results matched with the maximum spike rate recorded from large and medium spiking gustatory neurons in the medial sensilla cluster as well as large spiking neurons in the lateral sensilla cluster. The response strength of the neuron characterized by a medium-sized spike amplitude in the medial tarsal sensilla cluster was similar for 10 and 20 mM sinigrin and was significantly lower at 30 mM. This suggests that the response is saturated in the concentration range 10–20 mM and may imply that sinigrin occurs at such concentrations during tarsal drumming of the leaf surface of brassicaceous plants. The occurrence of glucosinolates in the waxy leaf cuticle of brassicaceous plants is controversial [33]. In one study on *Brassica nigra*, phloem sap of young leaves presented sinigrin concentrations greater than 10 mM [49]; however, no conclusive information on the concentration that the tarsal sensilla encounter during leaf contact is currently available. It has been suggested that during drumming the leaf cuticle is damaged and that the sensilla tips contact the leaf interior [33].

Our electrophysiological findings are largely in line with the previous findings on *P. brassicae*, but also in *P. rapae* and *P. napi*, in the sense that recordings of taste neuron activity in response to sinigrin consistently yielded two discrete spike classes characterized by different amplitudes [35–37,41]. Together, these results support the hypothesis that most sensilla in the fifth tarsomere of all three *Pieris* species contain at least two neurons that are responsive to sinigrin. Our data suggest that for both the large and the medium spikes of the medial cluster, as well as the large spikes of the lateral clusters, 10 to 20 mM sinigrin represents the optimal concentrations with regard to the spike rates. In addition, we found that in *P. brassicae*, the large-spiking neuron in medial cluster sensilla showed a low, but significant, response to sinigrin at a concentration of 20 mM, which has not been observed in the other *Pieris* species so far [36,41]. Moreover, recordings from neurons in the lateral cluster of *P. rapae* did not show any response to the aliphatic glucosinolate sinigrin, but only to the indolic glucosinolate glucobrassicin and the aromatic glucosinolate gluconasturtiin [37]. In our study on *P. brassicae* we did find a response of the large spiking neuron to sinigrin although only at a concentration of 20 mM. It is difficult to determine whether these different results represent species-specific responses or whether these are due to differences in the recording techniques or stimuli used. Notably, all studies have so far only found two to three neurons, while electron microscope images from *P. brassicae* indicate the presence of four chemoreceptor cells and one mechanosensory neuron in most sensilla [35]. Together, these findings suggest that *Pieris* females might still detect a greater variety of gustatory stimuli with their forelegs than we know of so far and that host-plant choice might ultimately depend on a combination of different gustatory channels [37]. Learning and memory formation experiments, like those reported here, might give additional insights into the valence associated with these different neuronal channels and their combinations for the female butterflies.

Glucosinolates, such as sinigrin, are well-known feeding and oviposition stimuli for *P. brassicae*. However, higher concentrations of these compounds can have detrimental effects on the performance of *Pieris* caterpillars [50], and it is, therefore, likely that different sinigrin concentrations carry different reward values for the female butterflies. The strength and reward value of the unconditioned stimulus and its role in learning and memory formation have been investigated in a number of studies on different insect species. For example, training the parasitoid wasps *Cotesia glomerata* and *Trichogramma evanescens* with a highly rewarding oviposition stimulus led to the formation of long-term memory, while a less rewarding experience only induced short-term memory formation [28]. Similarly, honeybees presented with odours coupled with different nectar rewards show higher recognition rates, and thus higher memory retention rates, for the odours associated with higher quality rewards [27]. We suggest that in *P. brassicae,* an increased gustatory response from the sinigrin-responsive neurons is indicative of a higher perceived reward quality, which subsequently leads to stronger memory retention. This is further supported by the low memory retention of butterflies conditioned on 30 mM sinigrin which correlated with the tendency for a low spiking rate when neurons were stimulated with 30 mM sinigrin. It can be speculated that plants with such high sinigrin concentrations might be less optimal hosts for the offspring of the female butterflies and that tarsal neurons would, therefore, signal a lower reward value during oviposition.

Changes in resource availability might push insect populations towards the use of novel food sources and oviposition sites, and therefore, the capacity to rapidly form associative memory with previously neutral stimuli might contribute to a higher fitness. In the current study, we demonstrated that there is a relationship between memory recall, oviposition site selection and the concentration of glucosinolates. The current climate and land use changes are likely to also affect the composition of glucosinolate-containing plants in the habitat of *P. brassicae* [51], which might require these butterflies to rapidly adapt their host-plant choice.

## 5. Conclusions

In the current study, we investigated the relationship between olfactory memory formation and the strength by which the unconditioned stimulus is detected in a butterfly. Our results show that the unconditioned stimulus concentration, which induced the strongest memory recall, matched the optimal range at which the oviposition stimulus is detected by specific populations of gustatory neurons. Furthermore, we demonstrate that memory formation and recall occurred in both lab and semi-natural conditions. Taken together, these findings highlight a potential role for olfactory learning and memory formation in butterflies in the context of host-plant selection and oviposition. Butterflies are among the best-studied insect groups with regard to their ecology and evolution, investigating the role olfactory learning plays during host-plant choice will help us to further understand how this insect group might respond to a changing environment.

## Data Availability

Behavioural and electophysiological data as well as R scripts are available at Dryad [52]. Supplementary material is available online [53].
